# Draft Genome Sequence of Pseudomonas chlororaphis subsp. *aurantiaca* ARS-38, a Bacterial Strain with Plant Growth Promotion Potential, Isolated from the Rhizosphere of Cotton in Pakistan

**DOI:** 10.1128/MRA.01398-19

**Published:** 2020-01-16

**Authors:** Samina Mehnaz, Andreas Bechthold, Harald Gross

**Affiliations:** a School of Life Sciences, Forman Christian College University, Lahore, Pakistan; b Department of Pharmaceutical Biology and Biotechnology, Institute of Pharmaceutical Sciences, University of Freiburg, Freiburg im Breisgau, Germany; c Department of Pharmaceutical Biology, Pharmaceutical Institute, University of Tübingen, Tübingen, Germany; Indiana University, Bloomington

## Abstract

Strain ARS-38 is a potential plant growth-promoting rhizobacterium that exhibits antifungal properties. Here, we report a 6.6-Mb draft genome, which gives insight into the complete secondary metabolite production capacity and reveals genes putatively responsible for its antifungal activity, as well as genes which contribute to plant growth promotion.

## ANNOUNCEMENT

As part of our ongoing efforts to investigate plant growth-promoting rhizobacteria (PGPR) (1–7), one of the authors (S.M.) isolated the strain Pseudomonas chlororaphis subsp. *aurantiaca* ARS-38 from the rhizosphere of cotton (8). It was shown that ARS-38, besides having antifungal properties, significantly increased root and shoot dry weights in wheat seedling growth assays (8), which is why this strain is considered a PGPR. Furthermore, chemical analyses proved ARS-38 to be capable of producing the metabolites indole acetic acid, hydrogen cyanide, lahorenoic acid (2), phenazines, a lipopeptide, and a hydroxamate-type siderophore (8). Therefore, we aimed to determine the whole-genome sequence of strain ARS-38 to reveal the genetic background of its antifungal capacity, as well as to provide a resource to study factors involved in plant association and potential biocontrol properties.

Strain ARS-38 was grown in 15 ml Trypticase soy broth (TSB) overnight at 25°C on a rotary shaker (120 rpm). For genomic DNA (gDNA) isolation, the Qiagen gDNA purification kit was used in combination with 100/G Genomic-tips according to the manufacturer’s protocol, except that for the bacterial lysis, the handled volumes were doubled, and incubation time at 50°C was prolonged until a clear lysate was obtained.

Next-generation sequencing was performed at 2,501× coverage using a PacBio Sequel platform with a 10-kb singleplex genomic library and 1 Sequel single-molecule real-time (SMRT) cell, obtaining 2,356,083 reads with a median read length of 7,023 bp. No quality filtering was conducted; however, subreads shorter than 50 bp were discarded. The remaining PacBio long reads were subsampled to a total of 3,000 Mbp and assembled using SMRTLink v6 and the Hierarchical Genome Assembly Process v4.0 (HGAP4.0) with default parameters and an expected 7-Mbp genome size, based on previously determined P. chlororaphis genome sizes (9–11). The draft genome of ARS-38 consists of 1 scaffold, a total of 6,615,046 bp, and a G+C content of 63.2%. Functional gene annotation using the NCBI Prokaryotic Genome Annotation Pipeline (PGAP v4.9) (12) identified 5,794 coding genes.

Automated secondary metabolism analysis using AntiSMASH v5.0.0 (13) predicted 17 biosynthetic gene clusters. Eleven of these matched known clusters for the biosynthesis of a pyoverdine (14), achromobactin (15), 3 homoserine lactones (16), phenazines (17), an arylpolyene (18), mangotoxin (19), *N*-acetylglutaminylglutamine amide (NAGGN) (20), and a resorcinol. The remaining clusters were predicted to encode 2 bacteriocins, 1 butyrolactone, 1 betalactone, and 2 nonribosomal peptide synthetase (NRPS)-based compounds.

### Data availability.

This whole-genome sequencing (WGS) project and the 16S rRNA gene sequence have been deposited at DDBJ/ENA/GenBank under the accession numbers CP045221 and KJ094432, respectively. The raw sequencing data are available from the Sequence Read Archive (SRA) under the accession number SRR10340775.
